# Evaluation of Near-Infrared Transparent Sealants for Occlusal Sealing: An In Vitro Study

**DOI:** 10.3390/ma18112421

**Published:** 2025-05-22

**Authors:** Camille Litzler, Lydia Vazquez, Clara Isabel Anton Y Otero, Ivo Krejci, Isaline Rossier, Marwa Abdelaziz

**Affiliations:** 1 Division of Cariology and Endodontology, CUMD—University Clinics of Dental Medicine, University of Geneva, Rue Michel-Servet 1, 1211 Geneva 4, Switzerland; camille.litzler@unige.ch (C.L.); clara.antonyotero@unige.ch (C.I.A.Y.O.); isaline.rossier@unige.ch (I.R.); 2 Department of Orofacial Rehabilitation, CUMD—University Clinics of Dental Medicine, University of Geneva, Rue Michel-Servet 1, 1211 Geneva 4, Switzerland; lydia.vazquez@unige.ch (L.V.); ivo.krejci@unige.ch (I.K.)

**Keywords:** caries, detection, diagnosis, DIAGNOcam, near infrared transillumination, occlusal sealing, sealant, dentistry, pedodontics

## Abstract

Background: This study aimed to analyze and compare the translucency and marginal adaptation of five resin-based materials used as occlusal sealants, both before and after simultaneous fatigue and thermocycling. Two null hypotheses were tested: (1) All tested materials allow the transillumination of sealed occlusal carious lesions. (2) There are no differences in marginal adaptation before and after simultaneous fatigue and thermocycling. Methods: Forty extracted human molars with early occlusal caries lesions were randomly divided into five equal groups. Near-infrared transillumination images of cleaned occlusal surfaces were captured before and after applying the following sealants: (I) OptiBond FL (adhesive alone), (II) OptiBond FL (primer and adhesive) (Kerr Corp., Brea, CA, USA), (III) Scotchbond Universal (3M, St. Paul, MN, USA) combined with OptiBond FL adhesive, (IV) Fissurit (VOCO GmbH, Cuxhaven, Germany), (V) Helioseal Clear (Ivoclar Vivadent AG, Schaan, Liechtenstein). A scanning electron microscope was used to assess marginal adaptation before and after simultaneous fatigue and thermocycling. The percentages of continuous margins (CMs) were quantified before and after the fatigue test and statistically compared (Shapiro–Wilk Normality test, two-way ANOVA with Fisher’s post hoc test). Results: Helioseal Clear and Fissurit were fully transparent under near-infrared transillumination. The percentage of closed margins significantly decreased after loading in one group: OptiBond FL primer application before adhesive application significantly reduced marginal adaptation. Conclusion: OptiBond FL (adhesive), Scotchbond Universal with OptiBond FL (adhesive), Fissurit, and Helioseal Clear provided excellent marginal adaptation. However, using OptiBond FL primer on enamel negatively impacted adaptation. Helioseal Clear and Fissurit, as transparent sealants, may allow lesion monitoring using an 850 nm transillumination camera.

## 1. Introduction

Dental caries represents a major burden for the health care system with about 2 billion people worldwide suffering from caries in permanent teeth, and 514 million children in primary teeth [1]. Although occlusal caries detection is usually achieved visually, near-infrared transillumination technology is increasingly applied for caries detection and monitoring [2,3]. Near-infrared transillumination cameras are diagnostic tools that allow the acquisition of near-infrared transillumination images by using an intraoral camera system including a light source with a wavelength between 700 and 1500 nm and a specifically developed software [3,4]. Identification and clinical staging of the presence, activity, and severity of dental caries is of paramount importance to determine treatment strategies and guide the choice between a non-invasive or a micro-invasive approach [5].

The sealing and infiltration of dental surfaces isolate the tooth from the oral environment, providing protection against demineralization and allowing health care providers to stop or slow down the caries process [6]. In addition, occlusal sealing aims to modify the surface of the retentive pits and fissures into smooth surfaces that are protected from bacterial colonization and exposure to fermentable substrates [7]. Occlusal sealing is an effective strategy not only as a preventive measure but also as a means to stop non-cavitated initial carious lesions [8]. Non-cavitated occlusal carious were successfully sealed with a transparent sealant for 44 months [9]. According to Fontana et al., sealants were 98% effective in preventing the progression of caries lesions in a population with a very high carious risk [9].

An ideal fissure sealant should be biocompatible, easy to handle, resistant to wear and fracture and have caries preventive effects. Cleaning the occlusal surface with hydrogen peroxide, pumice, air abrasion, and pretreatment with acid prior to sealant application has been suggested to improve the retention of the material on the tooth surface [10]. To date, there are numerous sealing products available on the market. Their composition varies between resin-based or glass-ionomer cement [11].

Resin-based sealants are mainly based on urethane dimethacrylate (UDMA) or bisphenol A–glycidyl methacrylate (Bis-GMA) monomers [12,13]. They have the advantage of a higher level of retention and better mechanical properties than glass ionomers [14]. Their disadvantage is polymerization shrinkage, potentially resulting in microleakage [15,16]. One of the most used resin-based unfilled sealants is Clinpro Sealant (3M, St. Paul, MN, USA). This sealant provides good retention, and it is colored when applied to the tooth and turns opaque white when light-cured [17,18]. The pink color makes it possible to visualize the location and the quantity of product that was applied but due to its opaque shade, it is not possible to monitor the evolution of the lesion under the product visually nor with transillumination.

Several resin-based (adhesive) materials, including bonding agents and sealants, are used for occlusal sealing. To enable effective monitoring of sealed occlusal surfaces using near-infrared transillumination, these materials must be sufficiently transparent. Before clinical investigations, comparative performance testing is essential due to the varied/different formulations of resin-based materials available on the market.

Monitoring the evolution of sealed carious lesions is important. Opaque sealants are easily applied and preferred by clinicians compared to transparent sealants because of the ease of application [19]. The disadvantage is the difficulty in determining visually and with transillumination whether the lesion underneath is progressing. Transparent sealants allow monitoring using clinical examination, laser fluorescence, or near-infrared transillumination technologies [20,21]. With near-infrared transillumination imaging technologies gaining ground in caries detection and monitoring, the use of transparent sealants is becoming attractive. There are only a few available transparent resin-based sealants on the market.

This study aimed to compare the translucency and marginal adaptation of five resin-based materials used as occlusal sealants, both before and after simultaneous fatigue and thermocycling. Two null hypotheses were tested: (1) All tested materials allow the transillumination of sealed occlusal carious lesions. (2) There are no differences in marginal adaptation before and after simultaneous fatigue and thermocycling.

## 2. Materials and Methods

### 2.1. Materials

The protocol that has been followed comes from a study conducted by Rodriguez Tapia, M.T., et al. [22]. Forty human molars with initial tooth decay were used in this study. The sample size calculation was based on the results of unpublished preliminary tests that detected an absolute difference of 8.45% in the continuous margins between the OBFL group (90.6%; SD 0.57) and Helioseal (82.15%; SD 7.06). With a power of 80% and a two-sided alpha error of 5%, we calculated a sample size of *n* = 8 specimens per experimental group. They were fixed in a mold made with a cold-curing resin (Technovit 4071, Kulzer, Wehrheim, Germany) [22]. They were then randomly assigned to five final study groups, each containing 8 samples. Initial documentation involved taking pictures of the occlusal surfaces with a Nikon camera D5300, objective 105 mm, and taking near-infrared transillumination images using the first generation of DIAGNOcam (1.006.5921 DC, KaVo, Biberach, Germany).

The teeth were sandblasted to remove debris and plaque with the MicroEtcher IIa (CD, Danville Materials, San Ramon, CA, USA) containing aluminum oxide, then etched with Ultra-Etch^TM^ (Ultradent Products, Inc., South Jordan, UT, USA) to improve sealant penetration [22]. These products are described in Table 1.

Then, the following materials were used: OptiBond FL (Kerr Dental, Brea, CA, USA) (adhesive only), OptiBond FL (primer and adhesive), Fissurit (VoCo GmbH, Cuxhaven, Germany), Helioseal Clear (Ivoclar Vivadent AG, Schaan, Liechtenstein), and Scotchbond Universal (3M, St. Paul, MN, USA) combined with OptiBond FL (adhesive). The materials’ compositions and manufacturers are shown in Table 2. The Helioseal and Fissurit sealants being unfilled or minimally filled and free of opacifiers exhibit excellent transparency to near-infrared light, while Scotchbond Universal and OptiBond FL adhesive contain silica fillers making them less transparent under near-infrared transillumination.

Between the initial and final treatments, the second-generation of near-infrared transillumination DIAGNOcam (1.013.5711 DC, KaVo, Biberach, Germany) came onto the market. To ensure the results were compatible with current clinical practice, we used the second-generation DIAGNOcam after loading. Both versions of the DIAGNOcam have the same wavelength but the image quality of the latest model has been improved. In addition, the second-generation DIAGNOcam takes three different pictures: infrared transillumination, fluorescence, and clinical pictures.

### 2.2. Methods

After the initial documentation, a sandblaster (MicroEtcher CD, Danville Materials, San Ramon, CA, USA) propelling aluminum oxide particles at 27 microns with a pressure of 2 bars was used to clean the occlusal surface of each tooth. Sandblasting was performed under a microscope with an 8× magnification (Leica MS6, Leica Microsystems AG, Heerbrugg, Switzerland) [22].

The dental grooves were conditioned with 35% phosphoric acid gel (Ultra-Etch^TM^) for 60 s. During etching, acid penetration was improved using an ultrasonic insert (ACTEON SATELEC, Merignac, France) for 30 s [22,23]. A study from Kersten et al. [23], showed that the penetration efficiency of phosphoric acid was enhanced thanks to the beneficial effect of an ultrasonic tip [23]. The teeth were then abundantly rinsed with water spray for at least 30 s and then dried with compressed air until the enamel appeared whitish and chalky. All samples were dried using 60% ethanol, followed again by compressed air spray [22,23].

**Figure 1:**
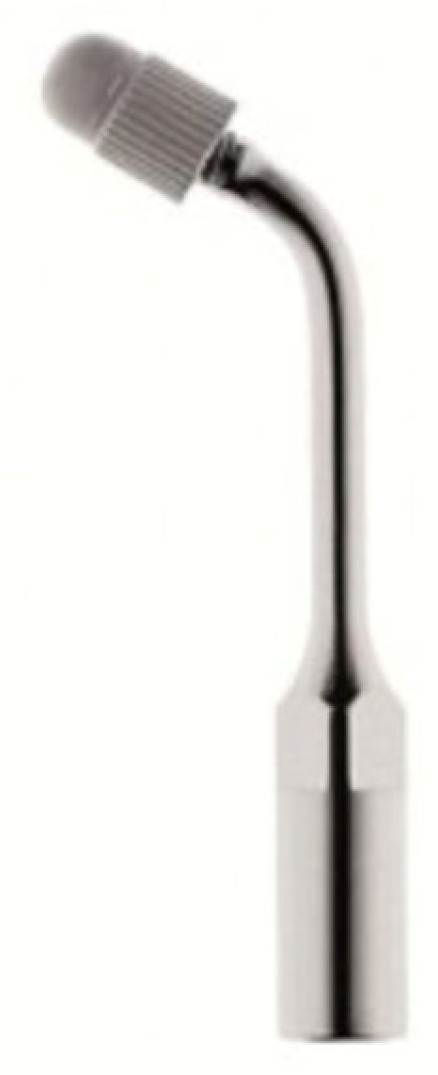
Ultrasonic insert used (ACTEON SATELEC, Merignac, France).

The materials described in Table 2 were then applied to the occlusal fissures with a dental probe [22]. An ultrasonic insert (ACTEON SATELEC, Merignac, France), covered with a round silicon tip (Figure 1) was gently positioned on the external surfaces of the tooth, avoiding contact with the pits and fissures [22].

The ultrasonic insert was used to ensure deep penetration of the resin in the grooves for 30 s. Under the microscope, we observed that during the application of the fissure sealant, air bubbles rose to the surface and were more easily eliminated with the gentle use of the ultrasonic insert. Applying ultrasonic vibrations to the tooth facilitates the displacement of air bubbles to the surface during sealant application [24]. A latency time of 30 s was allowed for the product to penetrate. Resins were then polymerized for 20 s mesial and 20 s distal by the LED LCU (LEDemetron II, Serial number 782033114, SDS Kerr, Middleton, WI, USA) [22].

Surfaces were coated with glycerin gel and the materials were polymerized for 60 s through the gel. This step aimed to avoid the inhibition layer formation on top of the resin material. After polymerization, the gel was removed with water spray [25].

A low-viscosity silicone impression (President Light Body, Coltene Whaledent, Alstätten, Switzerland) was used to replicate the epoxy resin occlusal surfaces (Epofix Kit, Struers, Rodovre, Denmark). The replicas were prepared and analyzed with the scanning electron microscope (XL 20, Philips, Eidhoven, The Netherlands). The micromorphology of the margins was evaluated as a percentage of the total quantity of margins analyzed according to the criteria of “continuous margins” or “marginal cracks” as described in previously published protocols [22,26].

After loading, we used the second-generation DIAGNOcam for transillumination and captured an image of the occlusal surface with a Nikon Camera D5300.

**Figure 2:**
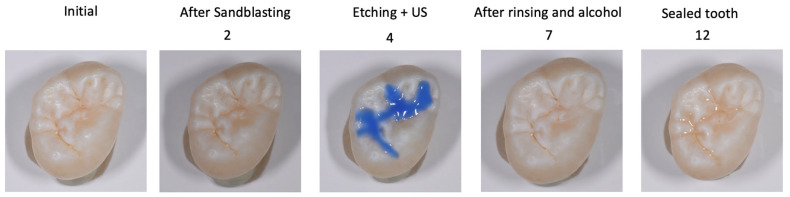
Steps of the protocol, the numbers correspond to Table 3. 2: After rinsing with water spray; 4: Etching with 35% orthophosphorique acid gel for 60s; 7: After drying with air and alcohol for 30s; 12: after rinsing of the glycerin gel with water spray and air-drying.

The steps of the protocol are shown in Figure 2 and explained in detail in Table 3.

### 2.3. Test Phase

Table 4 represents the final five groups and materials used for this study.

The sealed teeth were stored in the dark and in water at 37 °C for one week, then subjected to occlusal loading and thermocycling processes in a chewing simulator (SD Mechatronik, Feldkirchen, Germany) [26]. Thermocycling was performed using jets of water with alternating temperature from 5 °C to 50 °C, changing 3000 times with each cycle [22]. The immersion time for each temperature phase was 2 min [22]. The mechanical load, consisting of 1,200,000 load cycles, was transferred to the center of the occlusal surface with a frequency of 1.7 Hz and a maximum load of 49 N [22]. This load was applied via the lingual cusp of a molar in contact with the central sealing surface of the samples. After the cycle, another set of epoxy replicas was prepared and analyzed following the protocol described above [22]. For the evaluation of marginal adaptation, the replicas were subjected to a quantitative marginal analysis in a scanning electron microscope under ×200 magnification (Zeiss Gemini, Sigma 300 VP, Karl Zeiss Microscopy, Cambridge, UK) and a custom-made module programmed within image processing software (Marginal Analysis 1.0, University of Geneva, Geneva, Switzerland) performed by a single operator [22]

**Figure 3:**
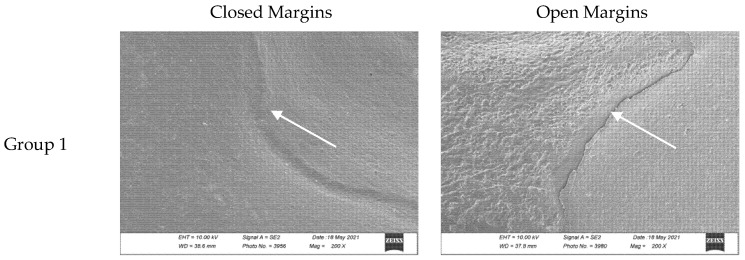

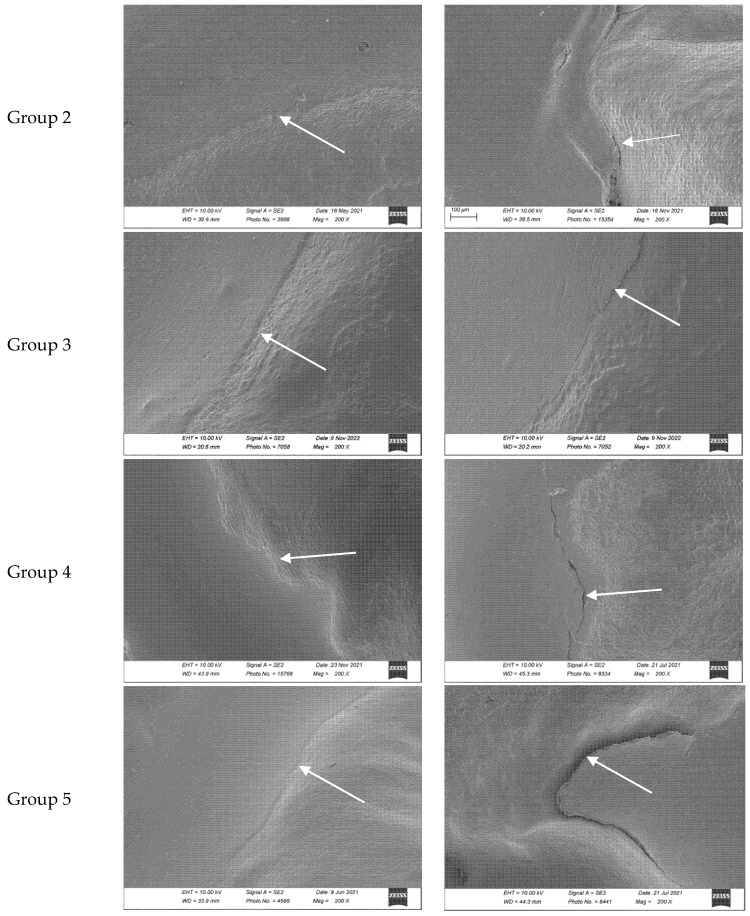
Closed and open margin examples for each group. Images were taken with a scanning electron microscope (using a 200× magnification) after the occlusal loading and thermocycling processes which simulate chewing cycles. Sealant margins are indicated with the white arrow. Groups 1 to 5 refer to Table 4.

Figure 3 shows the differences between a closed and an open margin in the scanning electron microscope images for each experimental group.

### 2.4. Statistics

All statistical tests were run with Stata/BE 17.0 2022 (StataCorp, College Station, TX, USA). The normal distribution assumption was checked by the Shapiro–Wilk normality test. Differences of continuous margins before and after loading were tested using a two-way ANOVA test followed by Fisher’s LSD post hoc test. The significance level was set to *p* < 0.05.

## 3. Results

### 3.1. Near Infrared Translucency

**Figure 4:**
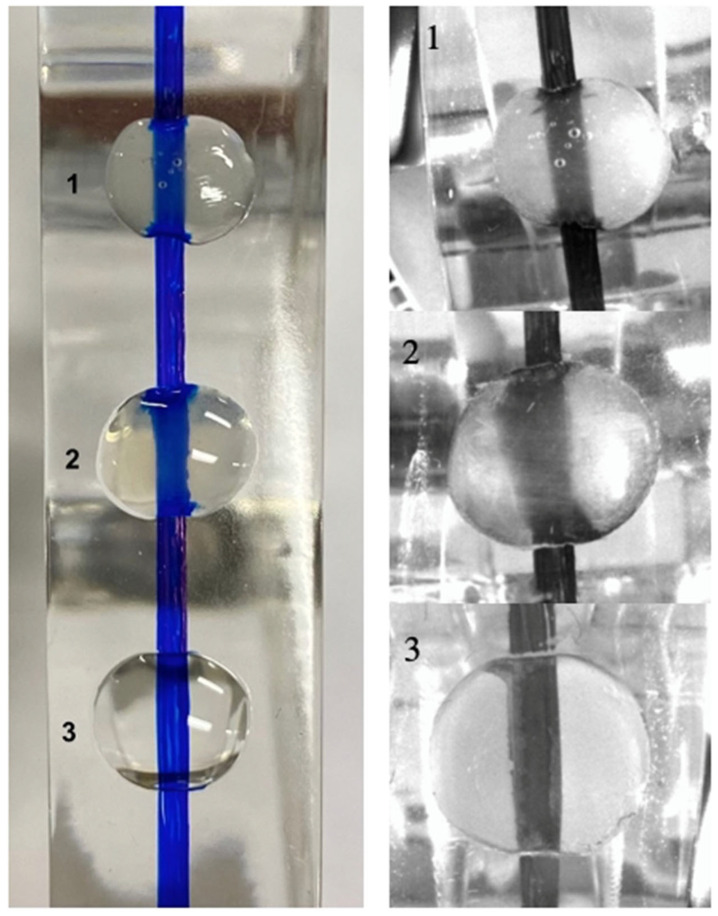
Clinical images of products on an epoxy block with a pen line compared to the second generation of DIAGNOcam infrared transillumination images. 1: Fissurit, 2: OptiBond FL adhesive, 3: Helioseal Clear with the second generation of DIAGNOcam. OptiBond FL is not fully transparent, and less so than the Fissurit and Helioseal Clear.

Helioseal and Fissurit sealants being unfilled or minimally filled and free of opacifiers exhibit excellent transparency to near-infrared light, while Scotchbond Universal and OptiBond FL adhesive contain silica fillers making them less transparent under near-infrared transillumination. To confirm the transparency of the products, transparent epoxy blocks were used to show the translucency of the materials. Images with the second-generation DIAGNOcam were repeated to compare the three products in Figure 4.

**Figure 5:**
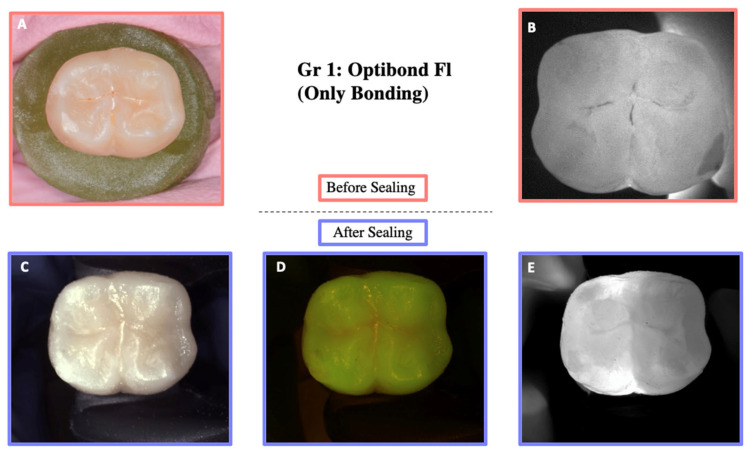
Illustration before sealing and after the loading of one of the teeth of Group 1. Images (**A**,**B**) were taken before sealing the teeth. Image (**A**) was taken with a Nikon D5300 camera (Nikon, Tokyo, Japan) and Image (**B**) with the DIAGNOcam first generation. After sealing the teeth, Images (**C**–**E**) were taken using the DIAGNOcam second generation. These images show that OptiBond FL is not transparent to transillumination.

**Figure 6:**
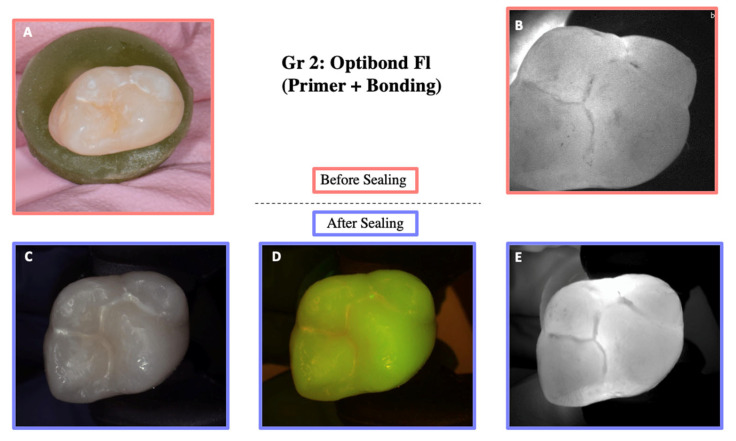
Illustration before sealing and after the cycle of one of the teeth of Group 2. Images (**A**,**B**) were taken before sealing the teeth. Image (**A**) was taken with a Nikon D5300 camera and Image (**B**) with the DIAGNOcam first generation. After sealing the teeth, Images (**C**–**E**) were taken using the DIAGNOcam second generation. These images show that OptiBond FL (primer and adhesive) is not transparent to transillumination.

**Figure 7:**
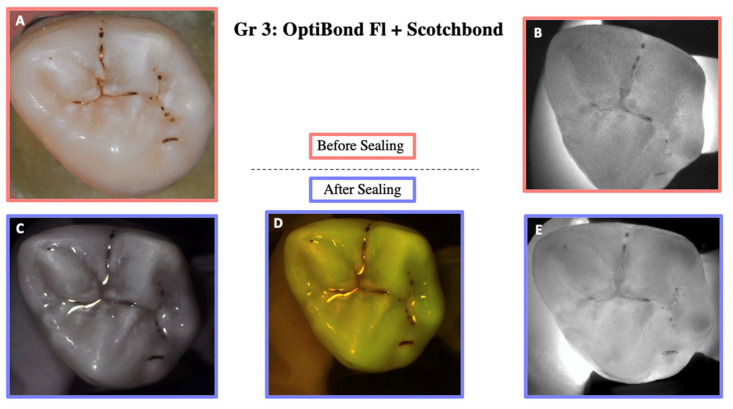
Illustration before sealing and after the cycle of one of the teeth of Group 3. Images (**A**,**B**) were taken before sealing the teeth. Image (**A**) was taken with a Nikon D5300 camera and Image (**B**) with the DIAGNOcam first generation. After sealing the teeth, Images (**C**–**E**) were taken using the DIAGNOcam second generation. These images show that OptiBond FL + Scotchbond Universal is not transparent to transillumination.

**Figure 8:**
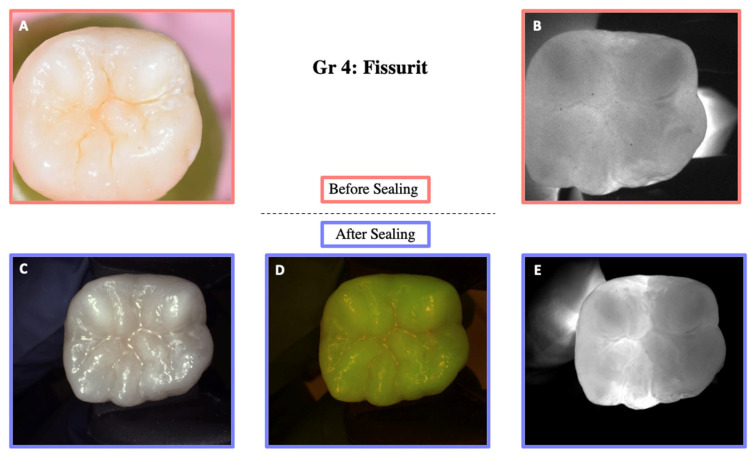
Illustration before sealing and after the cycle of one of the teeth of Group 4. Images (**A**,**B**) were taken before sealing the teeth. Image (**A**) was taken with a Nikon D5300 camera and Image (**B**) with the DIAGNOcam first generation. After sealing the teeth, Images (**C**–**E**) were taken using the DIAGNOcam second generation. These images show that Fissurit is transparent to transillumination.

**Figure 9:**
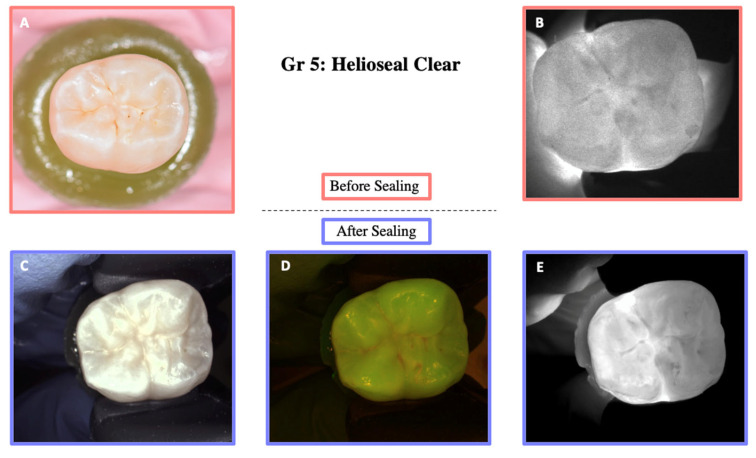
Illustration before sealing and after the cycle of one of the teeth of Group 5. Images (**A**,**B**) were taken before sealing the teeth. Image (**A**) was taken with a Nikon D5300 camera and Image (**B**) with the DIAGNOcam first generation. After sealing the teeth, Images (**C**–**E**) were taken using the DIAGNOcam second generation. These images show that Helioseal Clear is transparent to transillumination.

DIAGNOcam images obtained before sealing and after the test cycles are shown in Figure 5, Figure 6, Figure 7, Figure 8 and Figure 9.

The transillumination of the products was visually evaluated and is presented in Table 5.

### 3.2. Marginal Adaptation

Normality tests (Shapiro–Wilk) on the initial and final values for each group are described in Table 6. All *p*-values from the Shapiro–Wilk test were above the typical significance level of 0.05, suggesting that the second null hypothesis could not be rejected, and that the data were normally distributed.

The mean, standard deviation, and standard error of the mean value of the continuous margin were calculated for each group before and after the thermocycling and stress test.

**Figure 10:**
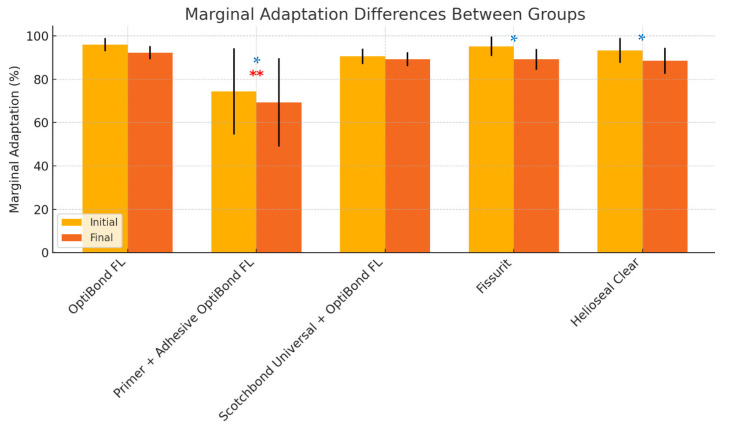
Differences between the average of closed margins in all groups before and after the mechanical and thermocycling test (significant differences marked with single asterisks (*) in blue for *p* < 0.05). Double asterisks (**) in red above Group 2 indicate its statistically significant difference compared to the other groups.

ANOVA analysis and multiple comparison analysis showed significant differences between initial and final marginal adaptation, except for Group 1 (OptiBond FL) and Group 3 (Scotchbond Universal + OptiBond FL (adhesive)) (see Table 7). However, when compared after loading to Group 2, which used the primer before the adhesive application (OptiBond FL, primer and adhesive), all groups showed significant differences (ANOVA *p* < 0.0061). Figure 10 and Table 7 and Table 8 provide a more detailed view of the results.

## 4. Discussion

With the introduction of new diagnostic and monitoring tools such as near-infrared transillumination allowing the detection and monitoring of early carious lesions, the need for transparent sealants becomes a high priority [2,27]. The first null hypothesis proposed was that all of the tested materials would allow the transillumination of sealed occlusal carious lesions. This null hypothesis had to be rejected because only Helioseal Clear and Fissurit were completely transparent to transillumination, whereas the others were not.

Some resin-based sealants tend to discolor due to dietary habits or external factors [28]; an assessment of color stability under simulated aging conditions could also be an interesting addition to future work.

Future studies comparing the findings with alternative diagnostic imaging techniques, such as laser fluorescence or optical coherence tomography, would be valuable.

Marginal adaptation is crucial for the clinical success of fissure sealing, as it reduces the risk of sealant loss and secondary caries. Several factors may have an impact on marginal adaptation. The first factor is the sealant material used. Research by Kantovitz et al. [16] evaluated the marginal adaptation of different sealing materials under thermal and chemical stress. They found that adhesive systems such as Single Bond had a significantly higher success rate (100%) in preserving seal integrity compared to traditional resin-based and glass-ionomer sealants [16]. Similarly, a study by Rodriguez Tapia et al. [22] confirmed the superior performance of OptiBond FL in terms of marginal adaptation and resistance to fatigue cracks under laboratory conditions.

In this study, OptiBond FL (adhesive) was chosen among adhesive systems because of its high filler load [29] and its recognized positive clinical record in its indication as an adhesive system [30]. As previous studies showed no differences between filled and unfilled sealants, it was speculated that a filled bonding could deliver similar results to a proper fissure sealant [31].

The second null hypothesis of this study stated that the marginal adaptation of the materials and their combination was the same, both before and after thermomechanical loading. Our study demonstrated that the adhesive sealing protocols, particularly using OptiBond FL (adhesive), Scotchbond Universal and OptiBond FL (adhesive), Helioseal Clear, and Fissurit, provided excellent marginal adaptation after thermocycling and mechanical stress tests. This second null hypothesis had to be rejected because primer application before OptiBond FL (adhesive) application significantly compromised marginal adaptation, as indicated by the lower percentage of continuous margins.

The negative impact of primer application on enamel adhesion was also reported by other authors who evaluated the long-term effect of dentin adhesives and application techniques on resin composite bond strength and marginal adaptation to enamel [32,33]. According to Frankenberger et al., the use of dentin adhesive systems showed no adverse effect on the long-term enamel bond strength and marginal adaptation. However, intensive rubbing of a primer decreased the bond strength and marginal adaptation over time [33]. Barreto et al. (2019) showed that using an excess of primer reduced the bond strength due to an unsatisfactory hybrid layer [32].

Another factor that may impact the marginal adaptation is the adhesive protocol. The adhesive protocol requires cleaning the surface from any contamination before sealing. The occlusal surface must be free of plaque, pellicle, calculus, and other contaminants, as they may impair the sealant’s diffusion and its ability to achieve intimate contact with the etched enamel [34]. Cleaning the surface with prophylactic pastes with a rotary bristle brush is frequently used by dental practitioners to remove debris from occlusal pits and fissures, even though this method seems to be less efficient in removing debris than sandblasting [35,36]. Additionally, surface moisture, variations in occlusal forces, salivary enzymes, or pH fluctuations could impact the results. Further clinical studies are needed to confirm these findings and explore the long-term effectiveness and caries-prevention benefits over time.

In our study, a sandblasting device was used prior to tooth sealing; in addition to cleaning the surface, air abrasion removed the outer enamel layer, which can be highly fluoridated [37] prism-less, and difficult to etch. Air abrasion did not eliminate the need for etching the enamel surface prior to sealant application [37]. Additionally, the particle size used during abrasion does not influence the bond strength between the sealing material and the occlusal surface [37,38].

Stavridakis et al. [36] evaluated the marginal adaptation of pit and fissure sealing and found that phosphoric acid etching significantly enhanced marginal adaptation compared to self-etching adhesive systems. This result reinforces the notion that the preparation method is a key factor in the performance of sealants [36]. Application of a strong acid increases the surface energy and the surface area and enhances surface porosity [39]. According to Hanning et al. [40], self-etching systems are not aggressive enough for enamel conditioning prior to fissure sealing. For this reason, in our study, 35% phosphoric acid etching was performed for 60 s. Etching fissures with 35% phosphoric acid for 60 s showed significantly less microleakage, longer tags, and better tag formation when compared to shorter etching times [41]. In our protocol, to increase the etching effect, the gel was agitated by short intermittent ultrasound vibration during application [23,42,43]. A water spray rinsing time of 30 s was respected because a sufficient rinsing time of the acid gel is important for an optimal adhesion to enamel [23]. However, future studies are needed to test other important characteristics such as exposure to an acidic environment [44] and color stability [45] in order to complete the knowledge about the behavior of sealant materials.

A limitation of the present study is the variation in tooth surface moisture before applying the sealant that could have affected the quality of adhesion and the marginal adaptation. Moreover, we tested only a limited number of materials in in vitro conditions. Clinical results may differ due to conditions such as saliva or blood contamination. Furthermore, an ultrasonic insert covered with a round silicone tip was used during the sealant application. Future studies could explore whether using different ultrasonic inserts may affect the setting of the sealant. Clinical studies using transparent sealing and diagnostic tools such as near-infrared transillumination for the monitoring of sealed fissures should be performed, as nowadays there are few clinical trials about this topic.

## 5. Conclusions

Helioseal Clear and Fissurit used as transparent sealants on initial carious lesion may allow easier lesion monitoring with an 850 nm wavelength transillumination camera while providing excellent quality sealing.

Marginal adaptation before and after loading with OptiBond FL (adhesive), Scotchbond Universal combined with OptiBond FL (adhesive), Fissurit, and Helioseal Clear provided excellent marginal adaptation. OptiBond FL primer applied on enamel seemed to hinder the quality of marginal adaptation.

## Figures and Tables

**Table 1:** Composition and manufacturers of the sandblaster and etching used.

Materials	Manufacturers	Batch No.	Composition	Protocol
MicroEtcher IIa	CD Dental	L1RLP	Aluminum oxide 27 microns	5 s with a distance of 10 mm
Etching Ultra-Etch	Ultradent	BKGJD	35% phosphoric acid	60 s activated with the ultrasonic insert

**Table 2:** Composition and manufacturers of the materials that were used.

Materials	Manufacturers	Batch No.	Composition
OptiBond FL	Kerr Dental	Primer: 9067698 Adhesive: 957693	Primer: HEMA ^1^, GPDM ^2^, MMEP ^3^, ethanol, water, initiators Adhesive: Bis-GMA ^4^, HEMA, GPDM, barium-aluminum, borosilicate glass, disodium hexaflurosilicate, fumed silica (48% filler)
Scotchbond Universal	3M	11004A	34% phosphoric acid, MDP ^5^, phosphate monomer, dimethacrylate Resins, HEMA modified, polyalkenoic acid, copolymer, filler, ethanol, water, initiators, silane
Fissurit	VoCo	2031227	Bis-GMA, UDMA ^6^, BHT ^7^, benzotriazolderiate, pyrogenic silicic acid
Helioseal Clear	Ivoclar Vivadent	W36096	Bis-GMA, triethylene glycol, dimethacrylate (99% weight), catalysts, stabilizers, and pigments (1%)

^1^ HEMA: Hydroxyethyl methacrylate; ^2^ GPDM: Glycerol Phosphate Dimethacrylate; ^3^ MMEP: Methacryloyloxyethyl Phosphate; ^4^ Bis-GMA: Bisphenol A–glycidyl methacrylate; ^5^ MDP: Methacryloyloxydecyl Dihydrogen Phosphate; ^6^ UDMA: Diurethane Dimethacrylate; ^7^ BHT: Butylated Hydroxytoluene.

**Table 3:** Protocol used in this study.

Protocol
Taking a picture of the occlusal surfaces with a Nikon camera D5300, objective 105 mm, and taking near-infrared transillumination images using the first-generation DIAGNOcam.
2. Sandblasting the surface with aluminum oxide at 27 microns to clean the surface.
3. Rinsing with water spray.
4. Etching with 35% orthophosphoric acid gel for 60 s.
5. Using the ultrasonic insert to allow penetration of the orthophosphoric acid in the grooves 30 s.
6. Rinsing with water spray.
7. Drying with air and alcohol for 30 s.
8. Material application with a dental probe.
9. Using the ultrasonic insert to promote the penetration of the sealant in the grooves for 30 s.
10. Polymerization 20 s occluso-mesial and 20 s occluso-distal with a 2000 mW/cm^2^ blue led light.
11. Coating of the occlusal surface with glycerin gel and polymerized for 60 s through the glycerin gel.
12. Rinsing off the glycerin gel with water spray and air-drying.
13. Taking a picture of the occlusal surfaces with a Nikon camera D5300, objective 105 mm, and taking near-infrared transillumination images using the second-generation DIAGNOcam.

**Table 4:** Final test groups’ repartition.

Group	Sealant Used
Group 1	OptiBond FL (adhesive only)
Group 2	OptiBond FL (primer and adhesive)
Group 3	Scotchbond Universal + OptiBond FL (adhesive)
Group 4	Fissurit only
Group 5	Helioseal Clear only

**Table 5:** The translucency or opacity of the sealing products to the DIAGNOcam is resumed in this table. The crosses (X) indicate whether the product is transparent or opaque. Fissurit and Helioseal Clear are transparent to transillumination, whereas the others are not.

Sealing Material	Transparent	Opaque
OptiBond FL (adhesive)		X
2. OptiBond FL (primer and adhesive)		X
3. Scotchbond Universal + OptiBond FL (adhesive)		X
4. Fissurit	X	
5. Helioseal Clear	X	

**Table 6:** Normality test results (Shapiro–Wilk). All the *p*-values were greater than 0.05, indicating that for all groups and time points (initial and final), the data did not significantly differ from a normal distribution.

Group	Initial	Final
OptiBond FL	*p* = 0.3817	*p* = 0.3712
2. OptiBond FL (primer and adhesive)	*p* = 0.2631	*p* = 0.4088
3. Scotchbond Universal + OptiBond FL (adhesive)	*p* = 0.9057	*p* = 0.5090
4. Fissurit	*p* = 0.3076	*p* = 0.3972
5. Helioseal Clear	*p* = 0.0748	*p* = 0.454

**Table 7:** The results of marginal adaptation (percentage of continuous margin) of different occlusal sealings. The median mean, minimum, maximum, standard deviation, and standard error of the mean were calculated for each group before and after the thermocycling and stress test. Groups 1 and 3 were similar before and after aging in contrast to the other groups that were significantly lower after aging.

	OptiBond FL (Group 1)		Primer + Adhesive OptiBond FL (Group 2)		Scotchbond Universal + OptiBond FL (Adhesive) (Group 3)		Fissurit (Group 4)		Helioseal Clear (Group 5)	
	Initial	Final	Initial	Final	Initial	Final	Initial	Final	Initial	Final
Mean	96	92.28	74.42	69.39	90.62	89.24	95.2	89.22	93.25	88.52
Median	96.73	92.23	78.87	74.06	96.29	90.48	95.58	87.43	90.45	88.24
Standard Deviation	2.97	3.08	19.96	20.38	3.54	3.17	4.46	4.78	5.70	5.95
Standard error of Mean	1.05	1.09	7.06	7.21	1.25	1.12	1.58	1.69	2.02	2.10
Minimum	90.84	88.67	36.8	35.99	85.51	84.71	86.33	80.93	81.72	79.28
Maximum	99.17	96.59	94.36	91.00	96.10	94.20	99.17	94.25	97.87	95.73
*p*-value	0.12		0.001		0.329		0.001		0.002	

**Table 8:** Tukey’s multiple comparisons test. Some comparisons showed significant differences (the one using the primer with the OptiBond FL and is showed with the asterisks **). Others showed no significant differences, meaning that the compared groups performed similarly. Scotchbond Universal: SBU.

Tukey’s Multiple Comparisons Test	Mean Diff.	95.00% CI of Diff.	Summary	Adjusted *p* Value
OptiBond FL vs. Primer + OptiBond FL	22.23	8.400–36.06	**	<0.001
OptiBond FL vs. Fissurit	1.928	−11.90–15.76	ns	0.994
OptiBond FL vs. Helioseal Clear	3.255	−10.58–17.08	ns	0.960
OptiBond FL vs. SBU + OptiBond FL	4.208	−9.622–18.04	ns	0.904
Primer + OptiBond FL vs. Fissurit	−20.30	−34.13– −6.472	**	0.001
Primer + OptiBond FL vs. Helioseal Clear	−18.98	−32.81– −5.146	**	0.003
Primer + OptiBond FL vs. SBU + OptiBond FL	−18.02	−31.85– −4.192	**	0.005
Fissurit vs. Helioseal Clear	1.327	−12.50– 15.16	ns	0.999
Fissurit vs. SBU + OptiBond FL	2.281	−11.55–16.11	ns	0.989
Helioseal Clear vs. SBU + OptiBond FL	0.9537	−12.88–14.78	ns	1.000

## Data Availability

The raw data supporting the conclusions of this article will be made available by the authors on request.

## Abbreviations

The following abbreviations are used in this manuscript:

MDPI	Multidisciplinary Digital Publishing Institute
UDMA	Urethane dimethacrylate
Bis-GMA	Bisphenol A–glycidyl methacrylate
HEMA	Hydroxyethyl methacrylate
GPDM	Glycerol Phosphate Dimethacrylate
MMEP	Methacryloyloxyethyl Phosphate
MDP	Methacryloyloxydecyl Dihydrogen Phosphate
BHT	Butylated Hydroxytoluene
SBU	Scotchbond Universal
4-MET	4-Methacryloxyethyl Trimellitate Anhydride
MDTP	Methacryloyloxydecyl Dihydrogen Thiophosphate
NS	Non-significant

## References

[1] Marcenes W., Kassebaum N.J., Bernabe E., Flaxman A., Naghavi M., Lopez A., Murray C.J. (2013). Global burden of oral conditions in 1990–2010: A systematic analysis. J. Dent. Res..

[2] Abdelaziz M., Krejci I. (2019). Longitudinal Caries Detection and Monitoring with Near Infrared Transillumination.

[3] Abdelaziz M., Krejci I. (2015). DIAGNOcam—A Near Infrared Digital Imaging Transillumination (NIDIT) technology. Int. J. Esthet. Dent..

[4] Sochtig F., Hickel R., Kuhnisch J. (2014). Caries detection and diagnostics with near-infrared light transillumination: Clinical experiences. Quintessence Int..

[5] Amaechi B.T. (2009). Emerging technologies for diagnosis of dental caries: The road so far. J. Appl. Phys..

[6] Chen Y., Chen D., Lin H. (2021). Infiltration and sealing for managing non-cavitated proximal lesions: A systematic review and meta-analysis. BMC Oral Health.

[7] Cvikl B., Moritz A., Bekes K. (2018). Pit and Fissure Sealants-A Comprehensive Review. Dent. J..

[8] Griffin S.O., Oong E., Kohn W., Vidakovic B., Gooch B.F., CDC Dental Sealant Systematic Review Work Group (2008). The effectiveness of sealants in managing caries lesions. J. Dent. Res..

[9] Fontana M., Platt J.A., Eckert G.J., Gonzalez-Cabezas C., Yoder K., Zero D.T., Ando M., Soto-Rojas A.E., Peters M.C. (2014). Monitoring of sound and carious surfaces under sealants over 44 months. J. Dent. Res..

[10] Beauchamp J., Caufield P.W., Crall J.J., Donly K., Feigal R., Gooch B., Ismail A., Kohn W., Siegal M., Simonsen R. (2008). Evidence-based clinical recommendations for the use of pit-and-fissure sealants: A report of the American Dental Association Council on Scientific Affairs. J. Am. Dent. Assoc..

[11] Da Silveira A.D., Borges B.C., de Almeida Varela H., de Lima K.C., Pinheiro I.V. (2012). Progression of non-cavitated lesions in dentin through a nonsurgical approach: A preliminary 12-month clinical observation. Eur. J. Dent..

[12] Ahovuo-Saloranta A., Forss H., Walsh T., Hiiri A., Nordblad A., Makela M., Worthington H.V. (2013). Sealants for preventing dental decay in the permanent teeth. Cochrane Database Syst. Rev..

[13] Feigal R.J. (2002). The use of pit and fissure sealants. Pediatr. Dent..

[14] Pardi V., Pereira A.C., Mialhe F.L., Meneghim Mde C., Ambrosano G.M. (2003). A 5-year evaluation of two glass-ionomer cements used as fissure sealants. Community Dent. Oral Epidemiol..

[15] Mehrabkhani M., Mazhari F., Sadeghi S., Ebrahimi M. (2015). Effects of sealant, viscosity, and bonding agents on microleakage of fissure sealants: An in vitro study. Eur. J. Dent..

[16] Kantovitz K.R., Pascon F.M., Alonso R.C., Nobre-dos-Santos M., Rontani R.M. (2008). Marginal adaptation of pit and fissure sealants after thermal and chemical stress. A SEM study. Am. J. Dent..

[17] Reddy V.R., Chowdhary N., Mukunda K.S., Kiran N.K., Kavyarani B.S., Pradeep M.C. (2015). Retention of resin-based filled and unfilled pit and fissure sealants: A comparative clinical study. Contemp. Clin. Dent..

[18] Kumaran P. (2013). Clinical evaluation of the retention of different pit and fissure sealants: A 1-year study. Int. J. Clin. Pediatr. Dent..

[19] Rock W.P., Potts A.J., Marchment M.D., Clayton-Smith A.J., Galuszka M.A. (1989). The visibility of clear and opaque fissure sealants. Br. Dent. J..

[20] Abdelaziz M., Krejci I., Perneger T., Feilzer A., Vazquez L. (2018). Near infrared transillumination compared with radiography to detect and monitor proximal caries: A clinical retrospective study. J. Dent..

[21] Celiberti P., Carvalho T.S., Raggio D.P., Mendes F.M. (2012). Influence of dental materials used for sealing caries lesions on laser fluorescence measurements. Lasers Med. Sci..

[22] Rodriguez Tapia M.T., Ardu S., Daeniker L., Krejci I. (2011). Evaluation of marginal adaptation, seal and resistance against fatigue cracks of different pit and fissure sealants under laboratory load. Am. J. Dent..

[23] Kersten S., Lutz F., Schupbach P. (2001). Fissure sealing: Optimization of sealant penetration and sealing properties. Am. J. Dent..

[24] Stewart C.W., Morrow B.R., Garcia-Godoy F. (2020). Evaluation of a novel instrument for placement of dental sealants. Am. J. Dent..

[25] Marigo L., Nocca G., Fiorenzano G., Calla C., Castagnola R., Cordaro M., Paolone G., Sauro S. (2019). Influences of Different Air-Inhibition Coatings on Monomer Release, Microhardness, and Color Stability of Two Composite Materials. BioMed Res. Int..

[26] Krejci I., Reich T., Lutz F., Albertoni M. (1990). An in vitro test procedure for evaluating dental restoration systems. 1. A computer-controlled mastication simulator. Schweiz. Monatsschr. Zahnmed..

[27] Mohamed Nur M.I.M. (2020). Near Infrared Transillumination for the Detection and Monitoring of Occlusal Caries: A Retrospective Clinical Study.

[28] Paolone G., Mandurino M., Scotti N., Cantatore G., Blatz M.B. (2023). Color stability of bulk-fill compared to conventional resin-based composites: A scoping review. J. Esthet. Restor. Dent..

[29] Peumans M., Kanumilli P., De Munck J., Van Landuyt K., Lambrechts P., Van Meerbeek B. (2005). Clinical effectiveness of contemporary adhesives: A systematic review of current clinical trials. Dent. Mater..

[30] Cehreli Z.C., Gungor H.C. (2008). Quantitative microleakage evaluation of fissure sealants applied with or without a bonding agent: Results after four-year water storage in vitro. J. Adhes. Dent..

[31] Boksman L., McConnell R.J., Carson B., McCutcheon-Jones E.F. (1993). A 2-year clinical evaluation of two pit and fissure sealants placed with and without the use of a bonding agent. Quintessence Int..

[32] Barreto S., Barbosa I., Pereira G., Dias C., Paulillo L. (2019). Effects of primer excess on marginal adaptation, nanoleakage and bond strength of adhesive systems after aging. Braz. J. Oral Sci..

[33] Frankenberger R., Kramer N., Petschelt A. (2000). Long-term effect of dentin primers on enamel bond strength and marginal adaptation. Oper. Dent..

[34] Symons A.L., Chu C.Y., Meyers I.A. (1996). The effect of fissure morphology and pretreatment of the enamel surface on penetration and adhesion of fissure sealants. J. Oral Rehabil..

[35] Burrow M.F., Burrow J.F., Makinson O.F. (2001). Pits and fissures: Etch resistance in prismless enamel walls. Aust. Dent. J..

[36] Stavridakis M.M., Favez V., Campos E.A., Krejci I. (2003). Marginal integrity of pit and fissure sealants. Qualitative and quantitative evaluation of the marginal adaptation before and after in vitro thermal and mechanical stressing. Oper. Dent..

[37] Courson F., Renda A.M., Attal J.P., Bouter D., Ruse D., Degrange M. (2003). In vitro evaluation of different techniques of enamel preparation for pit and fissure sealing. J. Adhes. Dent..

[38] Gray G.B., Carey G.P., Jagger D.C. (2006). An in vitro investigation of a comparison of bond strengths of composite to etched and air-abraded human enamel surfaces. J. Prosthodont..

[39] Handelman S.L., Shey Z. (1996). Michael Buonocore and the Eastman Dental Center: A historic perspective on sealants. J. Dent. Res..

[40] Hannig M., Grafe A., Atalay S., Bott B. (2004). Microleakage and SEM evaluation of fissure sealants placed by use of self-etching priming agents. J. Dent..

[41] Celiberti P., Lussi A. (2005). Use of a self-etching adhesive on previously etched intact enamel and its effect on sealant microleakage and tag formation. J. Dent..

[42] Duangthip D., Lussi A. (2003). Microleakage and penetration ability of resin sealant versus bonding system when applied following contamination. Pediatr. Dent..

[43] Duangthip D., Lussi A. (2003). Variables contributing to the quality of fissure sealants used by general dental practitioners. Oper. Dent..

[44] Poggio C., Dagna A., Chiesa M., Colombo M., Scribante A. (2012). Surface roughness of flowable resin composites eroded by acidic and alcoholic drinks. J. Conserv. Dent..

[45] Peker O., Bolgul B. (2023). Evaluation of surface roughness and color changes of restorative materials used with different polishing procedures in pediatric dentistry. J. Clin. Pediatr. Dent..

